# Improving coastal safety for international visitors to Australia

**DOI:** 10.1016/j.puhip.2025.100613

**Published:** 2025-05-10

**Authors:** William A. Koon, Robert W. Brander, Jasmin C. Lawes, Amy E. Peden

**Affiliations:** a School of Biological Earth and Environmental Sciences, University of New South Wales, Sydney, New South Wales, Australia; b Beach Safety Research Group, University of New South Wales, Sydney, New South Wales, Australia; c Surf Life Saving Australia, Sydney, New South Wales, Australia; d School of Population Health, University of New South Wales, Sydney, New South Wales, Australia

**Keywords:** Tourist safety, Drowning, Injury prevention, Travel medicine, Safety legislation, Environmental hazards, Marine tourism

## Abstract

**Objectives:**

International visitors are a high-risk group for drowning and other fatalities at Australian coastal locations due to lower visitation and familiarity than the resident population. This review of pre-COVID-19 (2005–2019) Australian international visitor coastal fatalities aimed to assess changes in mortality rates and evaluate differences between international visitor and resident death profiles to inform safety measures.

**Study design:**

Descriptive, retrospective epidemiological analysis.

**Methods:**

Analysis of unintentional coastal fatalities among international visitors to Australia from 2005 to 2019 was conducted using coronial data for fatalities and short-term visitor arrival data. Descriptive analysis comprised demographic, and incident-based variables, while cumulative (2005–2019) and annual fatality rates and 95 % confidence intervals per 100,000 short-term arrivals were calculated. Length of stay was incorporated into the risk measurement per 100,000 visitor-years. Joinpoint regression analysed trends in annual visitor coastal fatality rates.

**Results:**

Among coastal deaths 62 % were due to drowning; 12.8 % were international visitors; 7.83 residents died for each visitor fatality with an annual average of 22.5 visitor deaths. The cumulative visitor coastal fatality rate was 0.37 deaths per 100,000 international arrivals (95 %CI: 0.33–0.42), which decreased at a statistically significant level by an annual average of 5.8 % (95 %CI: 9.5 % to −1.9 %; p = 0.007) from 2005 to 2019. Visitors record an exposure-adjusted coastal fatality rate of 6.0/100,000 visitor-years. Visitor coastal deaths occurred in higher proportions in Queensland, at offshore locations, in more remote areas, while snorkelling, and during organised activities.

**Conclusions:**

Sustained efforts will require focus on high-risk visitor groups by diverse sectors including tourism, government, and water safety practitioners.

## 1 What this study adds

• A novel analysis of longitudinal trends in visitor coastal fatalities using Joinpoint regression is presented.
• This study is also the first to examine exposure-adjusted coastal fatality rates for international visitors, calculated as a rate per 100,000 visitor years.
• Differential risk profiles are highlighted to guide prevention efforts towards those at increased risk.

## 2 Implications for policy and practice

• Promotion of the coast is an important component of Australia's tourism campaigns, and thus maintaining visitor safety in these environments is vitally important.
• Significant reductions in coastal visitor fatalities across the study period suggests current interventions are effective and should continue to be implemented.
• A refinement in approach, or new approaches should be supported at a policy level to reduce risk among the high-risk cohorts identified in this study.

## 3 Introduction

Promotion of Australia's beaches and coast, and the strong attraction these areas represent as a tourist destination, is a primary driver of the country's visitor economy [1,2]. Prior to the Covid-19 pandemic, Australia's fourth largest export sector was international tourism, worth more than $45 billion AUD per year [3]. Coastal and aquatic experiences are central to most Australian holidays: six of the top ten Australian tourist attractions are related to aquatic or coastal experiences, an estimated 70 % of international visitors to Australia participate in coastal activities during their trip, and Australia's marine wildlife, remote beaches, and beaches near cities are considered world-leading [4]. National and state government tourism bodies also heavily promote Australia's beaches and coastal activities [5,6]. However, industry and government strategies around coastal and marine tourism mostly focus on economics, sustainability, and environmental impacts, with little mention of tourist safety or wellbeing [2,7].

Australia is generally considered to be a safe tourist destination, but coastal environments are inherently hazardous [8] and international visitor coastal safety has long been a priority of Australia's water safety sector [9]. Several Australian studies in the 1990's identified that international visitors (used synonymously with “tourists”) were particularly at risk of drowning in marine environments [[10], [11], [12]]. This finding was later re-affirmed when research identified drowning as the second leading cause of tourist deaths in the country [13,14] with coastal settings (beaches, ocean, and harbours) being the leading location for unintentional fatal drowning among international travellers [15,16]. At coastal sites, death or injury also occur from other non-drowning causes, such as surf zone impact injuries [17]. International tourist risk to these other types of coastal incidents has not been explored in the Australian context, but research from the United States indicates visitors are at greater risk [18].

Research from Australia has identified several reasons why international visitors are a high-risk group. These include variable swimming ability [19]; lack of local coastal knowledge [20] such as the ability to identify rip currents [21]; unfamiliarity with the Australian lifeguard safety practice of demarcating supervised safe recreational swimming areas with pairs of red and yellow flags [[21], [22], [23]]; seeking out and participating in risky activities such as snorkelling, body surfing and boating [24], but being unfamiliar with the environment and/or those activities [25]; and language barriers limiting one's ability to read and understand signs or safety information [22,26]. International research supports several of these findings, particularly that visitors have low ocean literacy and knowledge [27,28], ignore signs and lifeguard warnings [29,30], and express an overall relaxed demeanour towards safety while being on holiday [31].

International visitor safety at Australian waterways, including beaches and coastal areas, has been an explicit priority of the Australian Water Safety Strategy since its inception [9], and coastal safety interventions and efforts for visitors are wide ranging. Beyond supervised bathing, regulations and laws at local and state levels also serve to protect tourists (and residents) who engage in specific activities such as boating, snorkelling, and scuba diving [7]. The tourism industry in Australia has also successfully partnered with government and external stakeholders to co-develop codes of practice and best practice guidelines for operators [32,33] and codes of conduct for tourists [34] engaged in aquatic activities, providing recommendations and template procedures (e.g., checklists and audits) to ensure the safety and health of their clients and the protection of wildlife and the environment.

Lifeguards, legislation, and best practice guidelines for tourism operators are fundamental, but most efforts specific to international visitor coastal safety have focused on education, including in-person initiatives (e.g., stationing uniformed surf lifesavers in the arrival terminal of international airports to distribute safety brochures and talk with visitors, or an early morning “beach walk” program for Japanese tourists hosted and led by Japanese speaking surf lifesavers [19]). However, efforts are not standardised, nor universally available. Beach safety information, primarily focused on rip currents and promoting “swim between the flags”, is also readily available on many tourism/travel websites including the Australian government's Tourism Australia page [35], some foreign government Australian specific travel pages [36,37] and a variety of popular travel blogs and websites [38,39]. Various segments of the Australian travel industry also provide visitors with coastal safety information in strategic locations. Some airlines show beach safety information, such as a documentary about rip currents [40], on in-bound international flights; multilingual beach safety pamphlets and signs are available in many guest accommodations and at coastal locations [[41], [42], [43]]. Australian television also plays a role in educating international visitors about beach hazards [44].

To control the spread of Covid-19, the Australian government closed the international border to non-residents from March 20th, 2020, to February 21st, 2022, virtually eliminating short-term arrivals to the country for a two-year period. The pandemic has had drastic effects on the Australian tourism sector and visitor economy [45], which will likely take several years to return to pre-pandemic levels [46]. In the recovery phase, coastal and marine tourism is expected to increase as people place higher value on outdoor experiences in nature [7]. Nostalgia for travel and a drive to catch up on lost experiences due to the pandemic has prompted a tourism boom in other parts of the world that has caught some facets of the industry off guard [47,48]; with some predicting an accompanying increase in drowning risk [49]. In the recently released 2030 strategy for recovery of the tourism industry, the Australian government recognized the important role of aquatic and coastal experiences in the visitor economy, but discussions of safety focused only on aviation, older travellers, and Covid-19 [50] – not on drowning risk or other coastal safety concerns.

Prioritising coastal safety for international visitors must remain a major focus of Australia's marine and aquatic tourism sector. Although studies previously described have investigated various elements of tourist coastal safety in Australia [14,15,51], no study has specifically examined unintentional visitor fatalities that take place in coastal environments. Further, no study has evaluated if the decades long focus on international visitor beach and coastal safety has made a positive difference. To that end, this study serves to increase understanding of international visitor coastal fatalities in Australia, with a focus on examining changes in visitor coastal fatality rates. By providing information that will be of use to the water safety sector, as well as those working more broadly in public health, and the tourism industry, this research will help inform future efforts to keep tourists safe while they enjoy Australia's coast.

## 4 Methods

### 4.1 Study setting, period, and inclusion criteria

This descriptive, retrospective study is an epidemiological analysis of unintentional coastal fatalities among international visitors to Australia in the years 2005 through 2019. For this study, “visitors” refers to those who reside in another country and spent less than three months in Australia, generally tourists and those not working or studying. For comparison purposes, “residents” refers to those who usually live in Australia, including those on student and working holiday visas. In line with previous work, “coastal” refers to *the foreshore, seabed, large tidal bodies of water such harbours, bays, or inlets up to and including 12 nautical miles from the mean high tide line* [42,52]. We chose to analyse the years 2005 through 2019, purposefully excluding years impacted by the COVID-19 pandemic, as international border closures stopped incoming short-term arrivals. Coastal fatality cases were excluded from this analysis if: 1. they occurred in external Australian territories (e.g. Christmas Island) and the Torres Strait; 2. the death was intentional (homicide/suicide); 3. the deceased person was actively seeking asylum at the time of death; or 4. residency/visitor status could not be determined from the information available.

### 4.2 Data sources and variables

Fatality data used in this study were sourced from Surf Life Saving Australia's (SLSA) National Coastal Fatality Database, a coronial-based mortality index of all deaths occurring along Australia's coast as described in previous studies [52,53]. Researchers from SLSA maintain robust coastal fatality records based on information held in the National Coronial Information System (NCIS), which includes demographic information on the decedent and details on the nature of the fatality from coronial, autopsy, toxicology, and police reports. In the SLSA database, these data are supplemented with additional information from media reports and SLSA's SurfGuard Incident Report Database, when available. Data variable categorisation in this study follow previous work that uses the SLSA National Coastal Fatality Database [52]. The visitor status of the decedent is one of the variables captured in this database, allowing for definitive identification of visitors for individual cases.

Data on short-term visitor arrivals to Australia were sourced from the Australian Bureau of Statistics (ABS). The ABS data on short-term visitor arrivals, presents the number of overseas arrivals binned by estimated length of stay [54,55]. The ABS short-term arrivals data includes those intending to stay in Australia up to one year, however, to align with the SLSA National Coastal Fatality Database short-term visitor classification previously described, we restricted these data to short-term visitors with stays of less than three months.

### 4.3 Data analysis

We characterized visitor and resident coastal deaths by temporal, demographic, and incident-based variables using descriptive statistics including counts and percentages. To assess the visitor coastal fatality burden, we calculated cumulative (2005–2019) and annual fatality rates and 95 % confidence intervals per 100,000 short-term arrivals, and, to incorporate length of stay into the risk measurement, per 100,000 visitor-years. The person-time denominators in the visitor-year rates were derived from the length of time short-term arrivals spent in the country. The ABS data structure presents data by month, so for the month it was recorded, stays of under one week contributed 0.019 visitor-years, stays between one and two weeks contributed 0.038 visitor-years, and stays of two weeks to one month contributed 0.083 visitor-years. Stays between one and two months and two and three months also contributed 0.083 visitor years to the month it was recorded in, and 0.083 to the following month and following two months, respectively. For example, a three month stay arriving in December of 2005 would contribute 0.083 person-years to each month: December 2005, January 2006, and February 2006.

Trends in annual visitor coastal fatality rates were described and evaluated using joinpoint regression [56], which assesses for statically significant (p < 0.05) changes in rates over a study period and has previously been used to assess tourism mortality trends [57]. We used the Chi-squared test for independence to evaluate for differences between resident and visitor coastal fatalities by age group; sex; Australian state/territory of death; the remoteness location of death (*Offshore*, *Major Cities*, *Inner Regional*, *Outer Regional*, *Remote*, and *Very Remote)*; activity at the time of death, and company at the time of death (*Alone*, *With friends/family*, *With organised activity* (e.g., tour group), *With strangers* (e.g., bystanders that the decedent did not know), and U*nknown)*. A Bonferroni correction was applied where multiple levels of one variable were tested. Due to zero visitor fatalities in the 0–14 age group, this level was excluded from chi-squared analysis on age group.

Analysis was conducted using R (Version 4.2.0) and Tableau Desktop (Version 2021.3); joinpoint regression was conducted using the Joinpoint Regression Program (Version 4.9.1.0). Confidence intervals for fatality rates were calculated using Ulm's method in the epiR package (Version 2.0.48), assuming a Poisson distribution.

## 5 Results

**Fig. 1:**
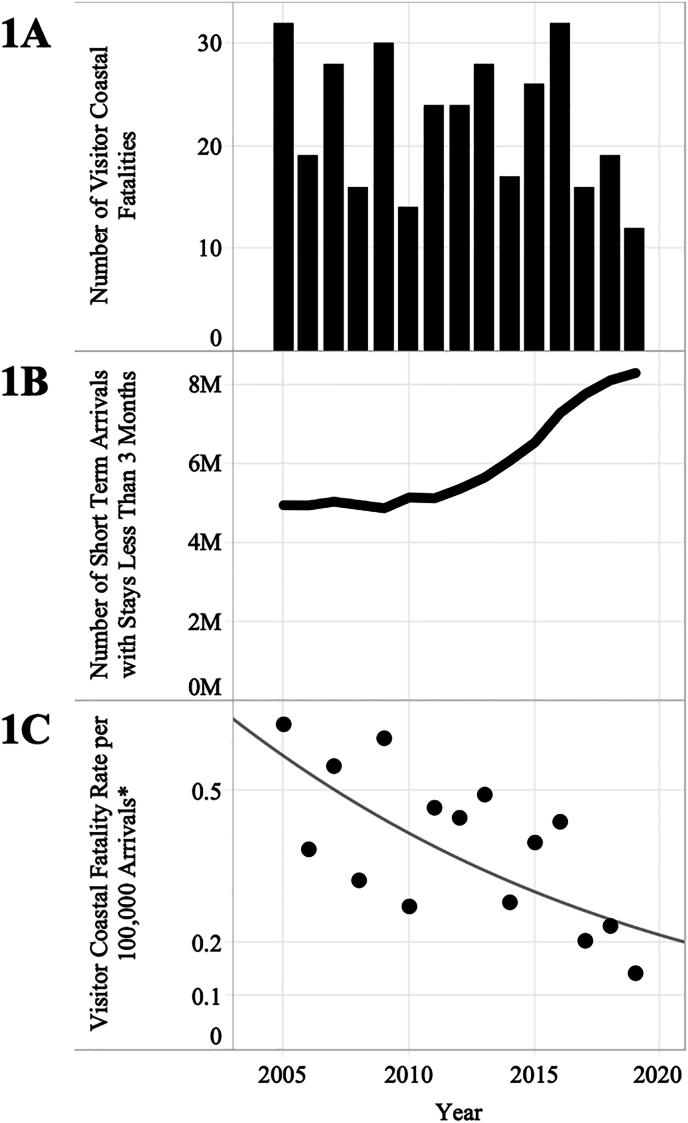
Short-term arrivals, visitor coastal fatality rates per 100,000 arrivals, and visitor coastal fatality deaths by year, 2005–2019. ∗ Trend for coastal visitor fatality rates reflects annual percent change of −5.8 % (95 %CI: 9.5 % to −1.9 %; p = 0.007).

From 2889 unintentional coastal deaths in the 2005–2019 study period, 2638 cases were analysed and 251 deaths were excluded (unconfirmed residency/visitor status [n = 133]; asylum seekers [n = 85]; external Territories and Torres Strait [n = 33]). Of the included cases, 12.8 % (n = 337) were visitors; 7.83 residents died at Australian coastal sites for each visitor fatality. An annual average of 22.5 visitor coastal deaths occurred [SD = 6.7] (Fig. 1A), most frequently (32.9 %) in the summer months of December, January, and February. Sixty-two percent (n = 1638) of coastal deaths were due to drowning, and 10.6 % (n = 173) of these, were visitors.

Compared to residents, visitor coastal deaths occurred in higher proportions in the state of Queensland, at offshore locations, in more remote areas, while snorkelling, and with organised activities (Table 1). While female visitors died at the coast in higher proportions than female residents (19 % vs. 13.6 %; *χ*^*2*^ = 9.36 (1), *P =* 0.002; Table 1), males still dominated both the visitor and resident fatalities: the male to female fatality ratio was 6.8 and 4.3 for residents and visitors, respectively. There was no difference in the age profile between visitor and resident coastal fatalities.

**Table 1:** Demographic, death location, and situational factors for coastal fatalities among residents and visitors, χ^2^ (df) P value, Australia, 2005–2019 (N = 2638).

Variable	Level	Total		Resident		Visitor		*χ2 (df) P value*
		N	%	N	%	N	%	
Total		2638	100	2301	87.2	337	12.8	–
Age group^a^								
	0–14	57	2.2	57	2.5	<5	<1	–
	15–29	503	19.1	439	19.1	64	19.0	0.001 (1) *P =* 0.96
	30–44	589	22.3	529	23.0	60	17.8	4.55 (1) P = 0.03
	45–59	706	26.8	612	26.6	94	27.9	0.25(1) P = 0.61
	60+	782	29.6	663	28.8	119	35.3	5.95(1) P = 0.014
	Unknown	<5	<1	<5	<1	<5	<1	–
Sex								
	Female	360	13.6	296	12.9	64	19.0	9.36 (1) *P =***0.002**
	Male	2278	86.4	2005	87.1	273	81.0	
State of death^b^								
	NSW	868	32.9	795	34.6	73	21.7	149.37 (1) *P* < **0.001**
	QLD	594	22.5	441	19.2	153	45.4	115.96 (1) *P* < **0.001**
	WA	447	16.9	375	16.3	72	21.4	5.36 (1) P = 0.02
	VIC	327	12.4	316	13.7	11	3.3	29.66 (1) **P < 0.001**
	SA	183	6.9	175	7.6	8	2.4	12.46 (1) **P < 0.001**
	TAS	125	4.7	114	5.0	11	3.3	1.86 (1) P = 0.17
	NT	94	3.6	85	3.7	9	2.7	0.89 (1) P = 0.39
Location of death^c^								
	Offshore	260	9.9	162	7.0	98	29.1	158 (1) P < **0.001**
	Major Cities	951	36.1	887	38.5	64	19.0	31.21(5) P < **0.001**
	Inner Regional	607	23.0	549	23.9	58	17.2	
	Outer Regional	471	17.9	409	17.8	62	18.4	
	Remote	181	6.9	155	6.7	26	7.7	
	Very Remote	165	6.3	136	5.9	29	8.6	
	Unknown	<5	<1	<5	<1	<5	<1	
Activity^d^								
	Boating & PWC	674	25.5	586	25.5	88	26.1	0.064 (1) P = 0.78
	Swimming/Wading	565	21.4	488	21.2	77	22.9	0.47 (1) P = 0.49
	Fishing & Rock Fishing	231	8.8	216	9.4	15	4.5	8.9643 (1) P = **0.002**
	Watercraft	195	7.4	181	7.9	14	4.2	5.9162 (1) P = 0.01
	Fall	167	6.3	157	6.8	10	3.0	7.3697 (1) P = 0.006
	Snorkelling	192	7.3	112	4.9	80	23.7	155.12 (1) P < **0.001**
	Scuba Diving	124	4.7	101	4.4	23	6.8	3.89 (1) P = 0.04
	Other	312	11.8	289	12.6	23	6.8	9.27 (1) P < **0.001**
	Unknown	178	6.7	171	7.4	7	2.1	13.39 (1) P < **0.001**
Company at time of death								
	Alone	557	21.1	522	22.7	35	10.4	112.3 (4) P < **0.001**
	With friends/family	1096	41.5	984	42.8	112	33.2	
	With organised activity	271	10.3	187	8.1	84	24.9	
	With strangers	142	5.4	125	5.4	17	5.0	
	Unknown	572	21.7	483	21	89	26.4	

Table footnotes: PWC = Personal Water Craft; Bonferroni adjustment applied (Age Group^a^ = 0.0125; State of death^b^ 0.0071; Location of death^c^ 0.025; Activity^d^ 0.005.

Visitor decedents were most frequently from Europe (43.6 %, n = 147), followed by Asia (35.6 %, n = 120) and North America (15.1 %, n = 51). Six countries had more than 20 decedents in the study period, representing 55.8 % of visitor all cases: United Kingdom (UK; 16.6 %, n = 56), United States of America (USA; 11.3 %, n = 38), China (9.2 %, n = 31), Philippines (6.5 %, n = 22), Japan (6.2 %, n = 21), and Germany (5.9 %, n = 20). People who died boating or operating a personal watercraft (PWC) (n = 88) came from 27 different countries, most frequently the Philippines (15.9 %, n = 14), followed by China (11.4 %, n = 10) and the UK (10.2 %, n = 9). Snorkelling deaths (n = 80) occurred among people from 24 different countries, most frequently the UK (17.5 %, n = 14) followed by the USA (16.3 %, n = 13) and Japan (11.3 %, n = 9). Swimming/wading deaths (n = 77) occurred among people from 29 different countries, most frequently the UK (20.8 %, n = 16) followed by China (10.4 %, n = 8) and tied for third rank, Canada, Germany, and the USA (6.5 %, n = 5).

In the years 2005–2019, there was a total of 90.3 million short-term arrivals to Australia with stays of under three months. Most of these visitors (63.4 %) stayed under two weeks, about a quarter (23.1 %) stayed between two weeks and one month, and 13.5 % stayed between one and three months. Annual arrival numbers remained mostly constant between 2005 and 2011 with an average increase of 1 % per year; but arrivals increased on average of 6 % per year between 2011 and 2019 for a total percent change of 54.6 % from 5.4 million in 2012 to 8.3 million in 2019 (Fig. 1B). The cumulative 2005–2019 visitor coastal fatality rate was 0.37 deaths per 100,000 arrivals (95 % CI: 0.33–0.42). Joinpoint regression results indicated a statistically significant decreasing trend in annual fatality rates per 100,000 arrivals: between 2005 and 2019, the rate decreased by an average of 5.8 % each year (95 %CI: 9.5 % to −1.9 %; p = 0.007; Fig. 1C).

Rates per 100,000 arrivals do not account for the varying time that visitors spend in the country. The 90.3 million short-term arrivals in the study period contributed a total of 5.6 million visitor-years, or 294 million visitor-weeks. Accounting for this exposure time results in a cumulative 2005–2019 coastal fatality rate of 6 deaths per 100,000 visitor-years (95 %CI: 5.3–6.6).

## 6 Discussion

This novel research investigated tourist safety on Australia's coast, with the two most important findings being that 1) visitor coastal death rates have decreased at a statistically significant level between 2005 and 2019, and 2) that the profiles of visitor and resident coastal fatalities diverged in several important areas. The statistically significant sustained decreasing trend for coastal fatalities indicates strategies currently employed are effective, particularly as a recent study found no clear decrease in overall international visitor death rates (from all locations, coastal and otherwise) in Australia [14].

In Australia, the percentage of drowning deaths that are visitors is relatively low compared to other locations with strong marine tourism sectors. In Greece, about one third of drowning deaths from all water sites were foreigners [58], and in Spain it was 19.2 % [59]. In Costa Rica, 38 % of coastal drowning deaths were international tourists, and the coastal drowning-specific fatality rate per 100,000 arrivals was higher than the all-cause coastal fatality rate from this study (1 per 100,000 arrivals in vs. 0.37 per 100,000 arrivals) [31]. In Kauai, Hawaii, USA, 73 % of drowning deaths were visitors, although there was no distinction between visitors from other countries vs. the US mainland [60]. While Australia's long-term commitment to coastal safety for international tourists may be partly responsible for these comparatively lower figures, variation in accessibility and tourist demographic profiles are also likely to play a role. Greece and Spain are easily accessible with short and cheap flights from other parts of Europe, as is Costa Rica and Hawaii from the continental United States and Canada. Comparatively, the long flight to, and subsequent accommodation in Australia is expensive, largely limiting international visitors to those with higher levels of disposable income. It is probable that higher income international travellers have participated in formal swim lessons and are more likely to stay in resorts or resort areas and participate in organised tours or activities, which in large part, driven by either legislation or motivated by genuine care for the client experience, have safety policies in place.

While Australian residents die on the coast in higher numbers (about 8 per visitor coastal death) and have more than double the coastal fatality rate per person, (0.81 per 100,000 residents [42] vs. 0.37 per 100,000 international arrivals), keeping international visitors safe on Australia's coast is paramount to the country's tourism recovery from border closures and evolution into a new age of international travel. Importantly, the decline in visitor coastal death rates shown in this study indicate preventive measures are effective. Although it is challenging to attribute reductions to any specific intervention, it would appear strategies such as improved dive safety practices [33], and visitor education about swimming at lifesaver patrolled beaches between the flags on inbound passenger flights, at the airport and in tourist accommodation [7] should continue to be supported. Beyond the legal duty of care businesses have for ensuring recreational water participants are not exposed to health and safety risks [33], safety is a major component of recreational quality for coastal tourists [61]. Genuine interest and meaningful engagement from government, businesses, the water safety and broader public health sector in keeping coastal visitors safe is key for re-establishing Australia's reputation as a world class tourist destination. This study provides a useful a starting point for managers and decision-makers charged with guiding this multifaceted process.

Tourists are not a uniform group, they vary in sensitivity to risk [62], and that the drivers of safety-related decision-making [63] can complicate risk communication and safety promotion efforts. Nevertheless, several of the results presented indicate opportunities for managers and decision makers to focus efforts and act.

While more international visitors died in major cities than other non-offshore remoteness categories, they died in greater proportions in *outer-regional, remote, and very remote* areas compared to residents (49 % vs. 33 %, respectively; Table 1). A variety of factors make water safety in rural Australia a complex issue [64], but two areas stand out as an obvious focus for visitor safety intervention: responsible promotion of remote beaches and the role of accommodation providers in remote coastal areas. Many remote beaches are hazardous and not patrolled by lifeguards or surf lifesavers [65,66] and active promotion of these ‘pristine’ and ‘untouched’ sites on social media has become a challenge [67]. Both government and tourism operators, including the communications apparatus of travel blogs, magazines, guides, and social media influencers, have an obligation to market a location's natural beauty responsibly. In Australia and other countries that have trained rescuers, that means only advertising and promoting beaches and other coastal locations with professional/trained lifeguards and/or dive/snorkel operators.

Snorkelling as a high-risk activity for international visitors in Australia is not a new concept, but this study re-affirms that continued focus by tourism operators and the water safety sector is required. Research from more than two decades ago identified snorkelling deaths as an issue of concern for international visitors and recommended prospective snorkellers should complete a medical fitness assessment [11] which is now classified as best practice [33], and in some states, codified with legislation [68]. The degree to which these practices are followed, however, has been identified as a challenge [7]. Compounding the issue further and linking to the previous discussion, snorkelling was identified as an activity that increases the likelihood international visitors would choose to visit remote locations in Australia [69]. Remote snorkelling, considering the distance from emergency services, is even more hazardous given the role that medical conditions play in snorkeller deaths [70,71]. Recent research from Hawaii has advanced the understanding of the previously unexplained medical aetiologies of snorkeller deaths, primarily, accidental or inadvertent aspiration and hypoxia resulting from acute negative pressure pulmonary edema [72]. Australia's tourism operator, dive medicine, and water safety communities should follow these developments closely as updates to policies and practices for snorkellers based on this emerging evidence may sustainably lower mortality risk while snorkelling.

### 6.1 Limitations

This research presents novel longitudinal outcomes that advance our understanding of international visitor mortality however, there remain some limitations. First, the calculated mortality rates underestimate the true burden of coastal international visitor deaths. In the person-based measurements per 100,000 international arrivals, it is important to note that not all international tourists visit the beach or coastal areas, meaning that a smaller denominator in the rate calculation would lead to larger overall rates. In the person-time measurements per 100,000 visitor-years, data from the ABS were grouped at pre-determined intervals, meaning that someone with a 10-day stay in Australia contributed 2 weeks (14 days) to the total person-time at risk used in the risk measurement. In short, the rate per 100,000 visitor-years presented here is conservative, and the real person-time measurement is likely higher. Second, there may be some misclassification in resident/visitor status, although unlikely, as information in the SLSA Database is triangulated using police and coroners’ reports. This study reports proportions of coastal fatalities by visitor region and country or origin, to assist in identification of priority groups for prevention efforts, but it does not report rates. This is worthy of future research, both in reporting these data but also in better understanding risk profiles, including activities undertaken and water safety behaviours, by visitors from different region/countries. Finally, while not a limitation, it is important to note that we chose to group international students with residents, not visitors. International students are generally in Australia for extended periods of time living, studying and working, more akin to a resident than a short-term international visitor.

## Ethics approval

This study was conducted with ethics approval from the Victorian Department of Justice and Community Safety Human Research Ethics Committee (CF/21/15898).

## Author contributions

WK conceptualized and designed the study and conducted formal analysis. WK drafted the original manuscript with input from AP and RB. JL was involved with data curation and editing the manuscript. All authors reviewed and approved the final manuscript.

## Sources of funding

This study received no funding. Author AEP receives funding from the [Australian] National Health and Medical Research Council (NHMRC) Emerging Leadership Fellowship (Grant ID: APP2009306). Open access funding support for the publication was provided by Surf Life Saving Australia.

## Declaration of competing interest

The authors declare the following financial interests/personal relationships which may be considered as potential competing interests: WK and RB have both received financial compensation from various local government and not for profit organisations for consulting projects related to beach safety. JL is employed by, and receives a salary from Surf Life Saving Australia, who are the custodians of the data used in this study.
